# Selflessness, Depression, and Neuroticism: An Interactionist Perspective on the Effects of Self-Transcendence, Perspective-Taking, and Materialism

**DOI:** 10.3389/fpsyg.2020.523950

**Published:** 2020-09-23

**Authors:** Christopher M. Wegemer

**Affiliations:** School of Education, University of California, Irvine, Irvine, CA, United States

**Keywords:** depression, neuroticism, selflessness, structure of the self, self-transcendence, perspective-taking, materialism, Buddhist psychology

## Abstract

Dominant theories of depression position self-concept as a central determinant of psychological functioning, but the relationship between the structure of self-concept and depression has not been extensively explored. The present study investigates the relationship between the structure of the self and psychopathological outcomes (depressive symptoms and neuroticism) with two methodological approaches. Using an established framework that draws insight from Buddhist psychology, the structure of the self is conceptualized in terms of selflessness and self-centeredness. Specifically, selflessness is construed as a multidimensional concept characterized by interdependence, outsider phenomenology, and impermanence. The three dimensions of the self were assessed at age 26 with inventories of self-transcendence, perspective-taking, and materialism, respectively (*N* = 814). First, a variable-centered approach was used to investigate potential interactions between the dimensions of selflessness. Self-transcendence negatively predicted depressive symptoms and neuroticism, whereas perspective-taking and materialism were positively associated with the outcomes. Self-transcendence moderated the relationship between perspective-taking and depressive symptoms. Perspective-taking was not statistically related to depressive symptoms for participants who exhibited higher levels of self-transcendence. The results clarify ambiguous associations between perspective-taking and depression found in previous research. Second, person-centered analyses were used to identify five profiles of self-structure: (1) *Selfless*, (2) *Selfless Materialist*, (3) *Interdependent Insider*, (4) *Self-centered Non-materialist*, and (5) *Self-centered*. As hypothesized, the *Selfless* cluster was associated with low levels of depressive symptoms and neuroticism, whereas the *Self-centered* cluster was associated with high levels. The profiles demonstrate the manifestation of several combinations of features of the self, which contributes to overall understanding of selflessness by complicating the traditional dichotomy between selflessness and self-centeredness.

## Introduction

The World Health Organization [WHO], (2018) has identified depression as the leading cause of disability worldwide. Globally, more than one in ten people experience depression in their lifetime (Lim et al., 2018). Despite advances in treatments, the incidence of depression has been increasing in recent decades (Weinberger et al., 2018). The rising prevalence has been accompanied by increased scholarly attention; research on depression has expanded substantially in recent years (Gotlib and Hammen, 2015). Psychological theories of depression have positioned self-concept as a key component (Beck, 1967; Kopala-Sibley and Zuroff, 2019). Broadening the perspectives of self-concept to emphasize the structure of the self may be a promising approach to develop a more comprehensive framework of depressive psychological functioning (Campbell et al., 2003; Stopa et al., 2010).

A variety of loosely overlapping literatures in self-psychology document associations between the structure of the self and depression. Studies of depression have invoked a range of structural features, such as compartmentalization (Showers, 1992; Brewin, 2006), self-concept differentiation (Donahue et al., 1993), self-complexity (Linville, 1987), self-concept clarity (Campbell et al., 1996; Bigler et al., 2001), and self-discrepancies (Higgins, 1987; Mason et al., 2019). The underlying logic is largely consistent across these studies; the structure of the self affects the availability and valence of self-knowledge, which in turn, influences negative self-evaluations in ways that are salient for psychopathologies. New perspectives of selflessness grounded in Buddhist psychology suggest that the structure of the self may be related to depression through processes other than self-evaluation. In the study presented below, I use a multidimensional model of the structure of the self (Dambrun and Ricard, 2011) to generate insights that may contribute to novel directions in the study of depression. First, I present the model and theoretical links between selflessness and psychological well-being. Then, I discuss the broader psychological and epistemological context that underlie arguments regarding the self. I conclude the literature review by describing the operationalization of the constructs, presenting hypotheses, and outlining an analytical plan. Ultimately, the present study seeks to advance understanding of both depression and selflessness.

## Selflessness and Depression

Utilizing an interdisciplinary foundation, Dambrun and Ricard’s (2011) Self-centeredness/Selflessness Happiness Model defines selflessness as a self-concept which contains structures and beliefs (implicit or explicit) that construe the self as a non-entity. The content, evaluation, and awareness of the self are not considered to be the primary determinants of selflessness. Rather, over time, self-beliefs shape the underlying structure of the self-concept, which change the function of the self across different contexts. Selflessness is distinguished from self-centeredness; these opposing self-concepts yield different psychological configurations of the self.

According to Dambrun and Ricard (2011), a “selfless” configuration of the self is characterized by features in three different dimensions: interdependence (experiencing strong connectedness and holding a weak distinction between the self and others, derived from Markus and Kitayama, 1991), “outsider” phenomenology (experiencing the self as an outsider looking at the self, based on Cohen et al., 2007), and impermanence (perceiving the flexibility of the self between experiences and contexts, drawing from Varela et al., 2016). In contrast, a “self-centered” configuration of the self has opposing qualities on these three dimensions: independence (holding the self as fundamentally separate from others with well-defined boundaries), “insider” phenomenology (dwelling within one’s experiences and giving exaggerated importance to the self), and permanence (perceiving a rigid and enduring sense of self over time and contexts). Selflessness and self-centeredness have been found to be separate factors (Dambrun, 2017), however, the present work advocates for a multidimensional empirical approach.

The model shares similarities with other frameworks of selflessness. Most notably, Wayment and Bauer (2008) distinguish between a “quiet ego” and a “noisy ego,” where individuals with a quiet ego are those who have transcended egotism and self-interest. The quiet ego is measured across four dimensions: non-defensive awareness, interdependence, perspective taking, and orientation toward personal growth (Wayment et al., 2015). Scholars have advanced similar models of selflessness (Levenson et al., 2005; DeCicco and Stroink, 2007; Metzinger, 2011; Shiah, 2016) and literature on selflessness has been closely linked to the study of wisdom (Levenson et al., 2005; Ardelt, 2008; Staudinger and Glück, 2011).

Two primary arguments motivate the present investigation of the relationship between selflessness and psychological well-being. First, Dambrun and Ricard (2011) make the case that functions of the self depend on different configurations self-structures. Their work linking self-centeredness with fluctuating happiness is extended by the current study to outcomes of depression and neuroticism. Second, the principles of Buddhist psychology underlying the present model maintain that self-centeredness is related to psychological suffering. This perspective is discussed in a subsequent section, whereas elaboration of the first argument is continued presently.

Dambrun and Ricard (2011) suggest that an independent, permanent self-structure that characterizes self-centeredness is associated with seeking pleasures and avoiding pain (that is, operating on the “hedonic principle”; Higgins, 1997), leading to cycles of satisfaction and dissatisfaction. In contrast, selfless individuals hold their self as interdependent and impermanent, which is associated with seeking harmony. Other models of selflessness have made similar assertions (Shiah, 2016). Empirical studies using Dambrun and Ricard’s (2011) model have found that selflessness predicts enduring happiness, whereas self-centeredness predicts fluctuating happiness (Dambrun et al., 2012; Dambrun, 2016, 2017; Hanley et al., 2017). More generally, behavior based on the hedonic principle has been related to negative affect and lower psychological well-being (Steger et al., 2008). Correspondingly, I anticipate a relationship between self-centeredness and depression.

Neuroticism has been strongly linked to depression (Kotov et al., 2010; Jeronimus et al., 2016). Neuroticism is a personality trait characterized by the tendency to experience negative affect (Ormel et al., 2013). Neuroticism has been construed as one of four self-evaluation dimensions, along with locus of control, self-efficacy, and self-esteem (Judge et al., 2002), all of which have been implicated in both self-centeredness and depression. Dambrun (2017) proposed that neuroticism is related to hedonic principle associated with self-centeredness and fluctuating happiness. I expect that neuroticism will be associated with both self-centeredness and depression.

## Differences in Psychological Traditions

Dambrun and Ricard’s (2011) conceptualization of selflessness is consistent with psychology’s recent shift toward incorporating multicultural perspectives. The movement of cognitive psychology in a more “positive” direction has led to the integration of Buddhist practices and ideas into an agnostic, scientific approach to well-being (Wallace and Shapiro, 2006; Segal and Teasdale, 2013; Shapiro et al., 2018). The theoretical foundation of Buddhist psychology is central to the current study’s investigation of depression and essential for understanding the current study’s relevance to literature on selflessness. Buddhism holds the self as illusory, interdependent, and impermanent, whereas Western psychology has historically viewed the self as a reified, independent, and enduring entity (Rubin, 1996; Miller, 2016); each perspective is briefly described below in relation to psychological well-being.

The International Classification of Diseases (ICD-10; World Health Organization [WHO], (2018) identifies self-esteem and self-reproach as two of the most important indicators of depression severity (Zimmerman et al., 2018). More generally, section III in the DSM-V describes two features of a non-pathological sense of self: (1) experiencing one’s self as a unique individual with clear boundaries between the self and others and (2) using one’s self as the basis for productively pursuing goals in life (American Psychiatric Association, 2013; Schmeck et al., 2013). Clinical psychology has traditionally focused on the development of a healthy sense of self through strengthening self-concept and correcting low self-esteem (Michalon, 2001; Mosig, 2006; Miller, 2016). Western psychology’s emphasis on a reified, independent self has roots in the ideas of the discipline’s foundational thinkers, such as Freud’s cultivation of “ego strength” (Freud, 1923) and Erikson’s normative identity as “selfsameness and continuity in time” (Erikson, 1959). The discipline has encouraged the development of high self-esteem (Baumeister et al., 2005; Singal, 2017) and even “healthy narcissism” (Kohut, 1971).

In stark contrast, Buddhist psychology denies the existence of a reified self (Dreyfus and Thompson, 2007), which is explicitly captured in Buddhism’s fundamental principles of *anatta*, *anicca*, and *dukkha*. Most directly, the concept of *anatta* (which translates as “non-self”) holds that there is no independent self. This is complemented by the second principle, *anicca* (“impermanence”), which acknowledges that there is no unchanging entity or essence of the self. The third characteristic of existence, *dukkha* (“suffering”), asserts that erroneously viewing of the self as independent and unchanging is a cause of suffering. The self is a “dynamically changing, self-organizing, multilevel, quasi-entity without sharp boundaries, and embedded in a causal thicket” (Galin, 2003). There is no independent being, only dynamic relationships (Garfield, 1995). The self is a process, useful for navigating the conventional world, but when it is mistakenly entified into what is perceived as a metaphysical object, suffering ensues (Galin, 2003). Buddhist psychology does not have a direct parallel to Western psychology’s conception of depression or its treatment. Rather, one of the primary Buddhist strategies to reduce suffering is the cultivation of selflessness through alignment with the reality that there is no independent and permanent self (Ekman et al., 2005; MacKenzie, 2016). Dambrun and Ricard’s (2011) work provides a conceptual bridge between Buddhist and Western conceptions of the self. Their model of the self can be operationalized using established measures in Western psychology to generate insights into psychopathologies, and reciprocally, such research may elucidate the function of selflessness in Buddhist psychology.

## Operationalizing Dimensions of the Self

In their model, Dambrun and Ricard (2011) described three self-structures associated with selflessness: interdependence, outsider phenomenological positionality, and impermanence. For self-centered individuals, these dimensions manifest as a bias toward treating self as a central point of reference, exaggerated sense of self-importance relative to others, and hedonic cycles of approaching pleasures and avoiding pain (Dambrun, 2017). The current work approximates these self-structures using inventories consistent with previous studies employing the model (Dambrun et al., 2012; Dambrun, 2017; Hanley et al., 2017), and more broadly, aligned with the field of self psychology (Wayment and Bauer, 2008; Baumeister, 2019).

First, interdependence is represented as self-transcendence. Self-transcendence is defined as decentering the self (Kohlberg, 1973), including the dissolution of obstacles to relating to others (Curnow, 1999), and has been conceptualized as a developmental process that captures changes in perspective of the self in relation to others (Levenson et al., 2001; Ardelt, 2008). The construct of self-transcendence closely resembles Dambrun and Ricard’s (2011) conceptualization of interdependence, construed as strong perceptions of connections with others and the external world that weakens the boundaries of the self.

Second, phenomenological positionality is operationalized as perspective-taking. Perspective-taking is defined as the ability and tendency to adopt the point of view of others (Davis, 1980). This construct was employed by Cohen et al. (2007) in their conceptualization of insider and outsider phenomenologies, from which Dambrun and Ricard (2011) derived their distinction. An exaggerated sense of importance given to the self manifests as lower levels of perspective-taking (Dambrun and Ricard, 2011).

Third, permanence (and the associated hedonic principle) are approximated using the construct of materialism. Materialism is defined as the importance of physical possessions for achieving desired psychological states or goals (Richins, 2004), which includes the centrality of material goods to one’s identity and beliefs regarding the happiness that material goods can provide (Richins and Dawson, 1992). Materialism has been construed in terms of construction and maintenance of the self (Shrum et al., 2013). Dambrun (2017) used materialism as an indicator of pleasure-seeking approach behavior in the hedonic process. The correspondence of impermanence and materialism is complex (Pace, 2013; Shrum et al., 2014), as impermanence is an expansive concept that extends beyond identification with material possessions. Nonetheless, materialism reflects a lower perception of impermanence of the self (Pace, 2013), and measuring manifestations of impermanence (e.g., materialism) may be a suitable strategy for partially capturing variance in impermanence. Materialism (and to a lesser extent, the other measures employed here) are approximate indicators of the underlying self-structures. The results of the analyses are interpreted with validity limitations in mind.

## Hypotheses and Analytical Plan

The current study is motivated by two research aims. First, I will investigate how self-transcendence, perspective-taking, and materialism interactively relate to depression and neuroticism. Second, I will examine the combinations of self-transcendence, perspective-taking, and materialism that emerge in a nationwide sample, then interpret these patterns in relation to selflessness and compare average levels of depression and neuroticism. I describe hypotheses regarding each research aim below, then present an analytical plan.

Regarding the first research aim, I expect that individuals low in self-transcendence and high in materialism will report greater depressive symptoms and neuroticism. Previous research has found that self-transcendence is negatively related to depression (Reed, 1991; Ellermann and Reed, 2001; Haugan and Innstrand, 2012) as well as neuroticism (Levenson et al., 2005). In contrast, materialism has been positively associated with depression (Kasser and Ryan, 1993; Dittmar et al., 2014) as well as neuroticism (Sharpe and Ramanaiah, 1999; Burroughs and Rindfleisch, 2002; Watson, 2015; Górnik-Durose, 2019).

Previous studies have yielded mixed results regarding the relationship between perspective-taking and depression (Schreiter et al., 2013). The present research may clarify the ambiguous findings. In contrast to previous studies on perspective-taking and depression, a consistently negative relationship has been found between perspective-taking and neuroticism (Lee, 2009; Melchers et al., 2016; Guo et al., 2018); I anticipate this association will also be present in the current study.

I expect to find interactions between self-transcendence, perspective-taking, and materialism in their associations with depression and neuroticism; this is the first study to include all three predictors simultaneously. Consistent with Buddhist psychology (Pace, 2013), Dambrun and Ricard (2011) suggest that these three features of the self interact through processes such as reification of the self and hedonic approach. Research on structure of the self has found moderation relationships between self constructs in their association with depression-related factors. For example, Stopa et al. (2010) found that an interaction between self-concept clarity and compartmentalization of the self partially accounted for anxiety. Also, mindfulness has been found to buffer against the negative effects materialism on depression, potentially implicating features of selflessness (Wang et al., 2017).

The ambiguity of past research on perspective-taking suggests that the relationship between perspective-taking and depression may be best understood through moderation tests (Schreiter et al., 2013; Powell, 2018). I hypothesize that perspective-taking will function differently depending on other structures of the self. Specifically, perspective-taking may be related to depressive symptoms for individuals who have an independent sense of self, but not for individuals who have an interdependent orientation. Eisenberg et al. (2010) emphasize that multiple emotional responses to perspective-taking are possible; for instance, some individuals may experience personal distress whereas others may experience empathic sympathy. In clinical studies of depressed patients, those who experienced personal distress tended to score higher on measures of perspective-taking (Thoma et al., 2011). Distress associated with perspective-taking is often linked to self-blame (O’Connor et al., 2007) and social withdrawal (Schreiter et al., 2013), which may exacerbate depression. Emotional regulation has been found to moderate the relationship between perspective-taking and depression (Powell, 2018); in a similar way, functions of the self may serve as a potential buffer. From Dambrun and Ricard’s (2011) theoretical perspective of selflessness, individuals with an outsider phenomenological position (characterized by high perspective-taking) and an independent, reified sense of self (low self-transcendence) may be more likely to experience personal distress (e.g., self-blame) that exacerbates depressive symptoms and behavior, whereas individuals with an interdependent sense of self (high self-transcendence) may be more inclined to feel prosocial emotions, such as empathic sympathy.

The second research aim will be fulfilled by exploring the combinations of features of the self that manifest in the present sample. Previous research has typically used variable-centered approaches that reduce the dimensions of selflessness to a selfless/self-centered dichotomy, sacrificing valuable nuance. Person-centered analyses using a large, diverse sample could clarify the characteristics and functions of selflessness. Further, specific combinations of self-structures are likely to be related to depression and these patterns may elucidate the interactions between the constructs established using variable-centered approaches. The usefulness of such multidimensional and person-centered analyses has been previously established in self-psychology (Diehl and Hay, 2011; Hanley et al., 2018). The underlying epistemology of person-centered analysis is consistent with the study of the psychology of the self; the individual is treated as an organized whole whose functions must be considered from a holistic and interactionist perspective (Bergman et al., 2003).

I predict that several conceptually meaningful profiles will emerge from clustering individuals based on self-transcendence, materialism, and perspective-taking. Dambrun and Ricard’s (2011) model suggests a minimum of two clusters representing selflessness and self-centeredness; individuals in the selfless category would have high self-transcendence, high perspective-taking, and low materialism, whereas individuals in the self-centered category would have the reciprocal of each characteristic. If such categories arise, I expect that cluster membership will be related to depression and neuroticism, with lower average levels of depression and neuroticism for individuals in the selfless cluster and higher average levels for individuals in the self-centered cluster. The observation of hypothesized moderation effects involving perspective-taking (described earlier) would suggest that at least two additional categories will be identified; one cluster may be high in self-transcendence and low in perspective-taking, whereas another may be low in self-transcendence and high in perspective-taking. Self-transcendence and perspective-taking are more directly tied to processes of reification of the self than materialism (Dambrun and Ricard, 2011) and may be more likely to drive cluster formation.

I will utilize two distinct methodological approaches to accomplish the two research aims. First, I will employ multiple regressions to test the relationship between features of the self (self-transcendence, perspective-taking, and materialism) and the outcomes of depressive symptoms and neuroticism. Interaction terms will be included to examine potential moderation among structures of the self, with attention to the ambiguous role of perspective-taking. Second, cluster analysis will be used to identify emergent inter-individual patterns in structures of the self. Cluster-level differences in depression and neuroticism will be tested with Multivariate Analysis of Covariance (MANCOVA), univariate Analysis of Variance (ANOVA), and chi square tests. The two complementary sets of analyses will identify potential linkages between the structures of the self and depression while clarifying the present model of selflessness (Dambrun and Ricard, 2011). Using both variable-centered and person-centered approaches in tandem provides complementary perspectives on interaction effects (Bergman and Magnusson, 1997) and may paint a more comprehensive picture of how features of the self associate with each other and psychopathological outcomes.

## Materials and Methods

### Participants

The National Institute of Child Health and Human Development Study of Early Child Care and Youth Development (NICHD SECCYD) is a longitudinal study that collected data on a cohort of children and their families between 1991 and 2018. Data collection began shortly after the birth of the study children in 10 locations around the United States: Little Rock, AR; Irvine, CA; Lawrence, KS; Boston, MA; Philadelphia, PA; Pittsburgh, PA; Charlottesville, VA; Seattle, WA; Morganton, NC; and Madison, WI. The children, now in adulthood, were participants in the present study. A full list of study items used in the current study is available in the Appendix. In the most recent wave of the NICHD SECCYD, 814 participants completed a survey at age 26. Participants were primarily White and middle class; sample statistics at recruitment and age 26 are described in Table 1. For a detailed description of the NICHD SECCYD and recruitment procedures, see the NICHD Early Child Care Research Network (2001); more information is available at https://www.icpsr.umich.edu/web/ICPSR/series/00233.

**TABLE 1:** Descriptive statistics of the NICHD SECCYD sample, at recruitment and at age 26.

		Recruitment (at 1 month)	Age 26
Gender			
	Female	48.3%	52.6%
Race/ethnicity			
	White	75.0%	81.0%
	Black	12.8%	8.6%
	Hispanic	6.6%	5.4%
	Other	5.6%	5.0%
Geographic location of birth			
	Little Rock, AR, United States	11.0%	5.9%
	Irvine, CA, United States	9.7%	11.8%
	Lawrence, KS, United States	9.8%	9.7%
	Boston, MA, United States	10.3%	10.6%
	Pittsburgh, PA, United States	9.0%	11.7%
	Philadelphia, PA, United States	10.0%	9.1%
	Charlottesville, VA, United States	10.0%	9.3%
	Seattle, WA, United States	10.2%	11.1%
	Morganton, NC, United States	10.6%	10.0%
	Madison, WI, United States	9.6%	10.9%
*N*		1,364	814

### Measures

#### Depressive Symptoms

The 20-item Center for Epidemiologic Studies Depression Scale (CES-D) was used to assess depressive symptoms (Radloff, 1977; Corcoran and Fischer, 1987). The well-established inventory measures somatic depressed affect (nine items, e.g., “I was bothered by things that usually don’t bother me”), positive affect (four items, e.g., “I felt hopeful about the future”), and interpersonal depressed affect (seven items, e.g., “I talked less than usual”). The CES-D was originally designed for use in non-clinical populations, but has also been employed in psychiatric settings (Corcoran and Fischer, 1987). Participants responded on a 4-point Likert scale describing how often they experienced each feeling or behavior, with scores of 1 corresponding to “Rarely or none of the time (Less than 1 day)” and scores of 4 corresponding to “Most or all of the time (5–7 days).” The items were averaged and the measure was standardized. The reliability of the scale was satisfactory (α = 0.94).

#### Neuroticism

A short version of the “Big Five” personality assessment containing 11 items was included in the survey (Gosling et al., 2003; Rammstedt and John, 2007). The short-form assessment was employed in a number of other large national surveys, such as the Panel Study of Income Dynamics (McGonagle and Sastry, 2014). Participants were asked to evaluate the extent to which they believed personality characteristics described them on a 4-point Likert scale from “Not at all” to “A lot.” Two items assessed neuroticism and were strongly correlated (*r* = 0.64). The items were averaged to create a single indicator of neuroticism and the measure was standardized.

#### Self-Transcendence

Six items from the Adult Self-Transcendence Inventory (ASTI) were used to assess self-transcendence (Levenson et al., 2005; Koller et al., 2017). Examples of items included non-attachment (e.g., “My happiness is not dependent on other people and things”), orientation of the self toward others (e.g., “I feel that my individual life is a part of a greater whole”), and peace of mind (e.g., “My peace of mind is not easily upset”). Participants were asked the extent to which they agreed with each statement on a 5-point Likert scale ranging from “strongly disagree” to “strongly agree.” One item exhibited low item-test correlation with the others and was removed (“I often engage in quiet contemplation”). The remaining items were averaged and the reliability of the scale was satisfactory (α = 0.74).

#### Perspective-Taking

Five items from the perspective-taking subscale of the Davis Interpersonal Reactivity Index (Davis, 1983) were used in the present study (e.g., “Before criticizing somebody, I try to imagine how I would feel if I were in their place”, “I believe that there are two sides to every question and try to look at them both”). Participants were asked the extent to which they agreed with each statement on a 5-point Likert scale ranging from “strongly disagree” to “strongly agree.” The items were averaged to create a composite measure. The reliability of the scale was satisfactory (α = 0.75).

#### Materialism

A 6-item short-form version of the Richins Material Values Scale was used to assess materialism (Richins and Dawson, 1992; Richins, 2004). The inventory measured three aspects of materialism: use of possessions to judge success (2 items, e.g., “The things I own say a lot about how well I’m doing in life”), centrality of possessions in a person’s life (2 items, e.g., “I like a lot of luxury in my life”), and the belief that the acquisition of possessions will lead to happiness (2 items, e.g., “I’d be happier if I could afford to buy more things”). Participants were asked the extent to which they agreed with each statement on a 5-point Likert scale ranging from “strongly disagree” to “strongly agree.” The six items were averaged and the reliability of the scale was satisfactory (α = 0.82).

#### Covariates

A number of variables were included as covariates in the analyses; the covariates included commonly used demographic controls (such as socioeconomic status and gender) as well as additional factors, chosen for theoretical reasons that suggested the constructs could potentially account for associations between features of the self and psychopathologies. For example, geographic location could act as a confound because it has been found to be related to both materialism (Ger and Belk, 1996) and depression (Magnusson, 2000). Accordingly, participant’s geographic location of birth was controlled.

Educational level was measured as the self-reported degree attained. Values ranged from 1 to 9, signifying “No high school diploma” to “Doctoral degree.” Annual income was assessed with one open-ended numerical item. Consistent with literature on depression, gender was treated as a binary construct. A dummy variable was used to indicate whether or not each participant was female. Lastly, a categorical variable was created to indicate whether the participant was identified as White, Black, Hispanic, or other.

### Missing Data

Four survey participants did not complete any of the items from the inventories of the current study and were excluded from data analyses. I employed single imputation on the remaining 810 cases. (Although multiple imputation is generally preferable, it was not appropriate for the current pattern-centered analyses; Basagaña et al., 2013). Sample sizes of each variable are presented in Table 2. The prominence of missing data ranged from 0% for gender, education level, and race/ethnicity to 5.5% for annual income. The imputation model included all study variables and covariates. Following established practices, values were imputed using the Markov Chain Monte Carlo method (MCMC; see Schafer, 1999).

**TABLE 2:** Correlations among study variables.

	1	2	3	4	5	6	7	8	9	10	11	12
(1) Self-transcendence	1											
(2) Perspective-taking	0.19*	1										
(3) Materialism	−0.11*	−0.11*	1									
(4) Depressive symptoms	−0.47*	−0.01	0.16*	1								
(5) Neuroticism	−0.48*	−0.01	0.12*	0.42*	1							
(6) Female	−0.10*	0.04	−0.09*	0.02	0.25*	1						
(7) White	−0.07*	−0.04	−0.11*	−0.12*	−0.02	0.02	1					
(8) Black	0.13*	0.03	0.09*	0.05	−0.03	−0.02	−0.63*	1				
(9) Hispanic	0.01	0.03	0.05	0.10*	0.04	−0.03	−0.49*	−0.07*	1			
(10) Other race/ethnicity	−0.04	0.00	0.04	0.04	0.04	0.02	−0.48*	−0.07*	−0.06	1		
(11) Education level	−0.05	0.03	−0.14*	−0.18*	−0.01	0.13*	0.16*	−0.14*	−0.10*	−0.01	1	
(12) Annual income	0.06	0.05	−0.02	−0.10*	−0.06	−0.01	0.04	−0.01	−0.03	−0.02	0.10*	1
Mean	3.45	3.84	2.87	1.74	2.70	0.53	0.81	0.09	0.05	0.05	5.24	51,073.34
*SD*	0.82	0.63	0.80	0.60	0.91	0.50	0.39	0.28	0.23	0.22	1.63	125,000
Min	1	1	1	1	1	0	0	0	0	0	1	0
Max	5	5	5	4	4	1	1	1	1	1	9	2,810,000
*N*	808	803	803	808	808	814	814	814	814	814	814	769

**For dichotomous variables, the mean represents the proportion of participants. ^∗^p < 0.05.**

## Multiple Regression Analysis

### Procedure

After conducting preliminary correlational analyses, I used ordinary least squares (OLS) regressions to test the relationship between the constructs representing the structures of the self (self-transcendence, perspective-taking, and materialism) and the outcomes of depressive symptoms and neuroticism. All predictors and outcomes were standardized prior to the analyses. Two-way and three-way interaction terms were created for self-transcendence, perspective-taking, and materialism to explore potential moderation effects. Lastly, I conducted simple slope analyses to aid the interpretation of the interaction.

### Results

I first investigated the zero-order correlations among the study variables (Table 2). The covariates were associated with outcomes of depressive symptoms and neuroticism. Participants were less likely to report depressive symptoms if they were white (*r* = −0.12, *p* = 0.032) with higher education levels (*r* = −0.18, *p* = 0.001) and greater income (*r* = −0.10, *p* < 0.001). Hispanic participants were more likely to report depressive symptoms (*r* = 0.10, *p* = 0.007). Female study participants were more likely to score higher on the measure of neuroticism (*r* = 0.25, *p* < 0.001).

Using OLS regression models, I examined the relationships between constructs representing structures of the self (self-transcendence, perspective-taking, and materialism) and depressive symptoms, controlling for gender, race/ethnicity, education level, annual income, and geographic site (see Table 3, model 1). Self-transcendence strongly predicted away from depressive symptoms (β = −0.50, *p* < 0.001), whereas perspective-taking (β = 0.10, *p* = 0.001) and materialism (β = 0.08, *p* = 0.012) were associated with depressive symptoms.

**TABLE 3:** OLS regression results, features of self-structure as predictors of depressive symptoms.

	Depressive symptoms, model 1				Depressive symptoms, model 2				Depressive symptoms, model 3			
	β	95% CI	*SE*	*p*	β	95% CI	*SE*	*p*	β	95% CI	*SE*	*p*
Self-transcendence	−0.50	[−0.56, −0.43]	0.03	<0.001	−0.50	[−0.56, −0.44]	0.03	<0.001	−0.49	[−0.55, −0.43]	0.03	<0.001
Perspective-taking	0.10	[0.04, 0.16]	0.03	0.001	0.11	[0.05, 0.17]	0.03	<0.001	0.11	[0.05, 0.17]	0.03	<0.001
Materialism	0.08	[0.02, 0.14]	0.03	0.012	0.08	[0.02, 0.14]	0.03	0.012	0.07	[0.01, 0.13]	0.03	0.020
Self-trans*Pers-taking					−0.07	[−0.13, −0.01]	0.03	0.019	−0.07	[−0.13, −0.01]	0.03	0.015
Self-trans*Mat					0.01	[−0.05, 0.07]	0.03	0.687	0.01	[−0.05, 0.07]	0.03	0.684
Pers-taking*Mat					0.00	[−0.06, 0.05]	0.03	0.890	−0.01	[−0.07, 0.05]	0.03	0.696
Self-trans*Pers-taking*Mat									0.04	[−0.01, 0.09]	0.03	0.123

**N = 810. Measures for depressive symptoms, self-transcendence, perspective-taking, and materialism were standardized. Controls included gender, race/ethnicity, education level, annual income, and geographic location. The adjusted R^2^ for model 1 was 0.30, F(18,791) = 18.85, p < 0.001. The adjusted R^2^ for model 2 was 0.29, F(21,788) = 16.50, p < 0.001. The adjusted R^2^ for model 3 was 0.31, F(22,787) = 15.89, p < 0.001*.*

Next, I tested for moderation in the relationship with depressive symptoms by adding interaction terms for self-transcendence, perspective-taking, and materialism to the regression model (see Table 3, models 2 and 3). The direct effects were nearly unchanged for self-transcendence (β = −0.49, *p* < 0.001), perspective-taking (β = 0.11, *p* < 0.001) and materialism (β = 0.07, *p* = 0.020). A significant interaction was observed between self-transcendence and perspective-taking (β = −0.07, *p* = 0.015).

**FIGURE 1:**
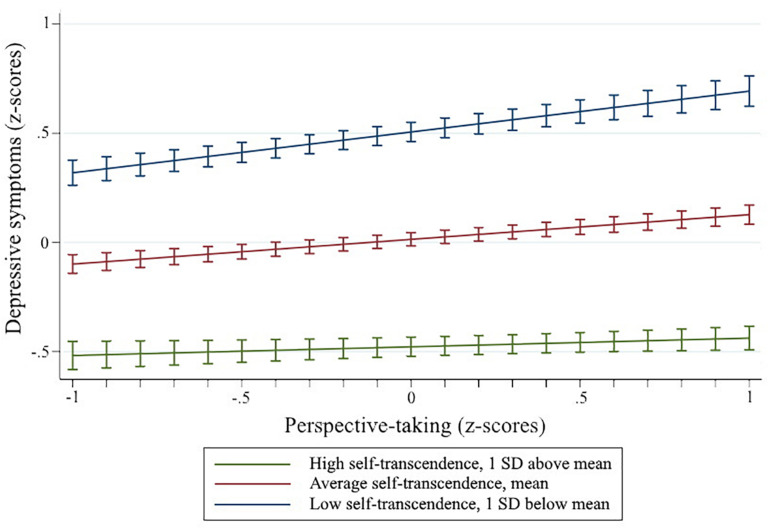
Moderation with perspective-taking as the independent variable. Error bars represent ± 1 standard error.

**FIGURE 2:**
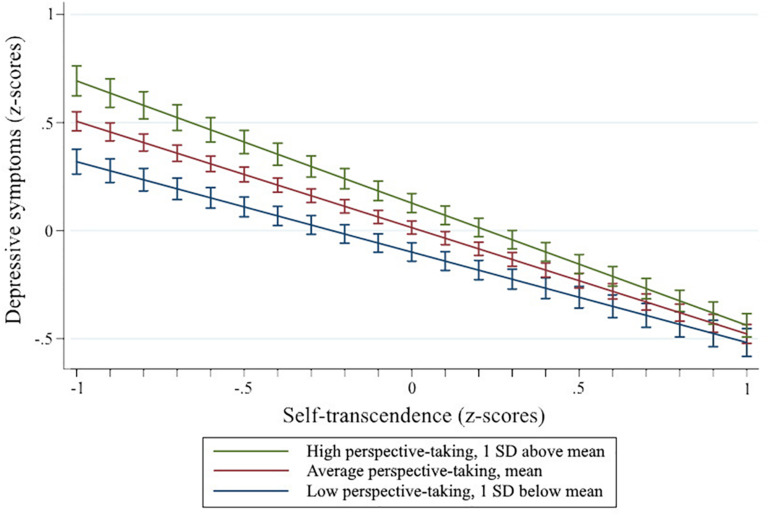
Moderation with self-transcendence as the independent variable. Error bars represent ± 1 standard error.

Following the protocol described by Aiken and West (1991), I examined the interaction between self-transcendence and perspective-taking via two sets of simple slopes analyses. First, I investigated perspective-taking as the independent variable and self-transcendence as the moderator (see Figure 1). The simple slope was statistically different from the horizontal with self-transcendence at one standard deviation below the mean (*b* = 0.34, *p* = 0.027), but not with self transcendence at one standard deviation above the mean (*b* = 0.07, *p* = 0.579). Second, I tested self-transcendence as the independent variable and perspective-taking as the moderator (see Figure 2). The simple slopes were statistically significant with both perspective-taking at one standard deviation below the mean (*b* = −0.24, *p* = 0.032) and one standard deviation above the mean (*b* = −0.56, *p* < 0.001). The slopes were statistically different (*p* = 0.003).

I then investigated the relationship between the self-structure predictors and neuroticism, controlling for all covariates (see Table 4, model 1). Paralleling the results for depressive symptoms, self-transcendence strongly predicted away from neuroticism (β = −0.47, *p* < 0.001), whereas perspective-taking (β = 0.08, *p* = 0.006) and materialism (β = 0.10, *p* = 0.002) were associated with neuroticism.

**TABLE 4:** OLS regression results, features of self-structure as predictors of neuroticism.

	Neuroticism, model 1				Neuroticism, model 2				Neuroticism, model 3			
	β	95% CI	*SE*	*p*	β	95% CI	*SE*	*p*	β	95% CI	*SE*	*p*
Self-transcendence	−0.47	[−0.53, −0.41]	0.03	<0.001	−0.47	[−0.53, −0.41]	0.03	<0.001	−0.46	[−0.53, −0.40]	0.03	<0.001
Perspective-taking	0.08	[0.02, 0.14]	0.03	0.006	0.09	[0.03, 0.15]	0.03	0.003	0.09	[0.03, 0.15]	0.03	0.003
Materialism	0.10	[0.04, 0.16]	0.03	0.002	0.09	[0.03, 0.16]	0.03	0.003	0.09	[0.03, 0.15]	0.03	0.003
Self-trans*Pers-taking					−0.04	[−0.10, −0.02]	0.03	0.144	−0.04	[−0.10, −0.02]	0.03	0.144
Self-trans*Mat					−0.01	[−0.07, 0.04]	0.03	0.619	−0.01	[−0.07, 0.04]	0.03	0.619
Pers-taking*Mat					0.00	[−0.06, 0.06]	0.03	0.871	0.00	[−0.06, 0.05]	0.03	0.871
Self-trans*Pers-taking*Mat									0.03	[−0.02, 0.09]	0.03	0.195

**N = 810. Measures for neuroticism, self-transcendence, perspective-taking, and materialism were standardized. Controls included gender, race/ethnicity, education level, annual income, and geographic location. The adjusted R^2^ for model 1 was 0.29, F(18,791) = 19.38, p < 0.001. The adjusted R^2^ for model 2 was 0.29, F(21,788) = 16.02, p < 0.001. The adjusted R^2^ for model 3 was 0.29, F(22,787) = 16.02, p < 0.001*.*

Lastly, I tested for moderation in the relationships between the self-structure predictors and neuroticism by adding interaction terms to the regression model (see Table 4, models 2 and 3). The direct effects were nearly unchanged for self-transcendence (β = −0.46, *p* < 0.001), perspective-taking (β = 0.09, *p* < 0.003) and materialism (β = 0.09, *p* = 0.003). None of the coefficients for the interaction terms were statistically significant.

### Discussion

A variable-centered approach was used to explore relationships between characteristics of the self, depressive symptoms, and neuroticism. Consistent with existing literature, self-transcendence was negatively associated with both depressive symptoms (Reed, 1991; Ellermann and Reed, 2001; Haugan and Innstrand, 2012) and neuroticism (Levenson et al., 2005). Findings regarding materialism were also aligned with prior research; materialism was associated with depressive symptoms (Kasser and Ryan, 1993; Dittmar et al., 2014) and neuroticism (Sharpe and Ramanaiah, 1999; Burroughs and Rindfleisch, 2002; Watson, 2015). The findings regarding perspective-taking diverged from existing scholarship and added nuance that may clarify the role of perspective-taking in depression.

A meta-analysis investigated eleven studies that assessed the relationship between perspective-taking and depression (Schreiter et al., 2013); 8 of the studies yielded a negative association between perspective-taking and depression, whereas the remaining 3 found no association. A more recent study by Tully et al. (2016) further complicated the ambiguous literature, concluding that individuals with high levels of perspective-taking had greater depressive symptoms. Using the same indicator as these studies (Davis, 1983), I found that perspective-taking was positively associated with depressive symptoms. The association was only present when controlling for self-transcendence; regression models that did not contain self-transcendence yielded no relationship between perspective-taking and depressive symptoms. (There was no risk of multicollinearity between self-transcendence and perspective-taking; *r* = 0.19, *p* < 0.001). The results suggest that the extent to which an individual perceives their own tendency to adopt the point of view of others is associated with depression, after accounting for whether individuals see themselves as interdependent with others. The current work is aligned with recent studies that have attempted to elucidate the ambiguous relationship between perspective-taking and depression by testing potential moderators (Tully et al., 2016; Powell, 2018).

The current study is the first to uncover an interaction between self-transcendence and perspective-taking, which represents a novel perspective in the depression literature. There are multiple possible interpretations of the moderation effect. The results of the simple slopes analyses provide evidence that self-transcendence buffers against the negative effects of perspective-taking; perspective-taking is related to depressive symptoms for individuals with low self-transcendence, but not for individuals with high self-transcendence. When testing perspective-taking as a moderator, the results of the simple slopes analyses were less straightforward. Self-transcendence was negatively related to depressive symptoms for all individuals regardless of perspective-taking, although the difference in slopes suggest the depressive symptoms outcome was more sensitive to differences in self-transcendence for individuals with higher levels of perspective taking. Interpreting self-transcendence as the moderator of the relationship between perspective-taking and depressive symptoms has theoretical precedent. Individuals’ experiences of perspective-taking vary; possible emotional reactions include prosocial sympathetic states and personal distress (Eisenberg et al., 2010). Similarly, and consistent with Dambrun and Ricard’s (2011), differences in self-structure and self-other orientation may determine the functionality of perspective-taking (Batson, 2009). Perhaps relatedly, emotional regulation has been found to moderate the relationship between perspective-taking and depression (Powell, 2018). Future studies may clarify the association of perspective-taking and depression by exploring potential linkages between self-transcendence and emotional regulation.

## Cluster Analysis

### Procedure

I conducted person-centered analyses using k-means clustering techniques through a multi-step process (Vargha et al., 2015). *K*-means clustering is an effective method to maximize within-group homogeneity and between-group heterogeneity without the same shortcomings as traditional dendrogram approaches to clustering (Bergman et al., 2003). I employed ROPstat software (v2.0) in accordance with established guidelines (Vargha et al., 2015). The three self-structure indicators previously described (self-transcendence, perspective-taking, and materialism) were used as clustering indicators, each of which were unstandardized.

Prior to constructing clusters, I conducted outlier tests using residue analysis (see Bergman et al., 2003, pp. 111–114); results suggested that no cases needed to be removed. Next, I employed hierarchical cluster analysis (HCA) using Ward’s method based on squared Euclidian distances to obtain a preparatory classification. Initial cluster centroids obtained from HCA were used as non-random starting points for iterative *k*-means clustering. Relocation of cases between clusters based on reduction of the overall error sum of squares for the profile solution allowed for greater within-cluster homogeneity (Vargha et al., 2015). Identification of the most appropriate solution was guided by fit indices and theoretical expectations. I used *k*-means clustering to construct nine sequential sets of cluster solutions, with profiles consisting of two to ten clusters. A scree-type plot was used as an aid to compare the change in explanatory power and parsimony of each solution (Peck et al., 2008).

### Results

**FIGURE 3:**
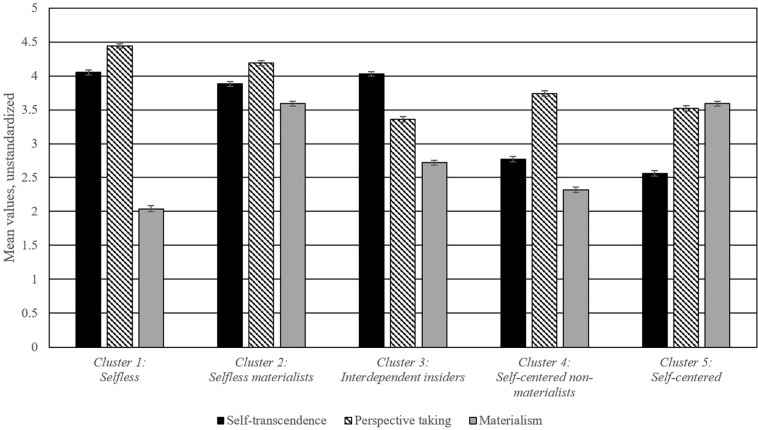
Results of the cluster analyses. Error bars represent standard error (±1 SE above and below the cluster means).

A five-cluster solution emerged as the most suitable profile, explaining 58.2% of the variance in the cluster factors. The Homogeneity Coefficient of each cluster (HC; the average distances of between all pairs of cases within each cluster) indicated that the cases within clusters were sufficiently homogenous (HC < 1; Vargha et al., 2015). Each of the five clusters were approximately the same size. Compared to other potential profiles, the explained error sum of squares ranged from 28.9% for the two-cluster solution to 72.4% for the 10-cluster solution. Following the procedure established by Bergman et al. (2003), a sudden jump in the explained error sum of squares indicated the bifurcation of two distinct clusters and suggested that five clusters represents both a parsimonious and conceptually meaningful solution. The explained error sum of squares of the five-cluster configuration surpassed the recommended 50% threshold (Milligan and Cooper, 1985). The next best configuration was a four-cluster solution, but this was unacceptable because the explained error sum of squares was substantially lower than the five-cluster solution (52.0%) and the homogeneity coefficients were undesirably large (HC > 1). The four-cluster solution was also less theoretically salient than the five-cluster solution. In sum, the five-cluster solution emerged as the best configuration describing the data and was used for further investigation. For each cluster, unstandardized mean-level values of each indicator were displayed graphically (see Figure 3) and standardized z-scores were presented numerically (see Table 5).

**TABLE 5:** Mean-level differences in cluster characteristics.

	Cluster 1: Selfless		Cluster 2: Selfless materialist		Cluster 3: Interdependent insider		Cluster 4: Self-centered non-materialist		Cluster 5: Self-centered			
	*M*	*SD*	*M*	*SD*	*M*	*SD*	*M*	*SD*	*M*	*SD*	*F*(*p*)	η^2^
Clustering indicators												
Self-transcendence	0.74_*a*_	0.60	0.52_*b*_	0.61	0.72_*a*_	0.54	−0.84_*c*_	0.61	−1.09_*d*_	0.62	366.97 (*p* < 0.001)	0.65
Perspective-taking	0.94_*a*_	0.65	0.56_*b*_	0.73	−0.76_*c*_	0.81	−0.15_*d*_	0.80	−0.50_*e*_	0.85	136.08 (*p* < 0.001)	0.40
Materialism	−1.03_*a*_	0.68	0.89_*b*_	0.58	−0.18_*c*_	0.60	−0.69_*d*_	0.59	0.90_*b*_	0.60	339.75 (*p* < 0.001)	0.63
Outcomes												
Depressive symptoms	−0.36_*a*_	0.82	−0.19_*a*_	0.85	−0.37_*a*_	0.72	0.24_*b*_	1.00	0.64_*c*_	1.15	36.89 (*p* < 0.001)	0.15
Neuroticism	−0.38_*ab*_	0.92	−0.13_*a*_	1.01	−0.41_*b*_	0.92	0.34_*c*_	0.91	0.56_*c*_	0.84	35.66 (*p* < 0.001)	0.15
*N*		149		166		167		164		164		

**All variables are standardized. Means within a row with the same subscript are not significantly different at p < 0.05, as determined by Tukey’s HSD tests. Univariate ANOVAs were employed, with between-group df = 4 and within-group df = 805*.*

I assigned the following names to the five clusters: (1) *Selfless*, (2) *Selfless Materialist*, (3) *Interdependent Insider*, (4) *Self-centered Non-materialist*, (5) *Self-centered*. Clusters were given names consistent with contemporary labeling practices (Wormington and Linnenbrink-Garcia, 2017); the unstandardized raw scores were first taken into account, then relative comparisons between clusters using *z*-scores were used to refine the labels consistent with the theoretical model employed (Dambrun and Ricard, 2011). Each of the clusters is briefly described below.

#### Selfless

This cluster is characterized by high levels of self-transcendence and perspective-taking and low levels of materialism. The values for self-transcendence and perspective-taking are the highest among all the clusters (although the *Interdependent Insider* cluster has statistically equivalent values for self-transcendence). The values for materialism are the lowest among all clusters.

#### Selfless Materialist

Similar to the Selfless cluster, this cluster is characterized by above-average levels of self-transcendence and perspective-taking. However, in contrast to the Selfless cluster, the levels of materialism are high. This is the only cluster to have above-average levels on all three indicators. The values of materialism are highest among all the clusters, statistically equivalent with the Self-centered cluster. The present cluster (Selfless Materialist) and the Self-centered cluster are the only two that have above average values for materialism.

#### Interdependent Insider

This cluster is characterized by high levels of self-transcendence, low levels of perspective-taking, and slightly below-average levels of materialism. The values of self-transcendence are the highest among all the clusters (along with the statistically equivalent Selfless cluster), whereas values of perspective-taking are the lowest among all the clusters.

#### Self-Centered Non-materialist

All measures are below average for this cluster. Self-transcendence and materialism are low, whereas perspective-taking is slightly below average. This is the only cluster to have below-average levels on all three indicators.

#### Self-Centered

This cluster is characterized by low levels of self-transcendence and perspective taking and high levels of materialism. The values of self-transcendence are lowest among all the clusters. The values of materialism are the highest among all the clusters (statistically equivalent to the *Selfless Materialist* cluster).

A Multivariate Analysis of Covariance (MANCOVA) indicated that the clusters differed from each other on outcomes of depressive symptoms and neuroticism, after controlling for gender, race/ethnicity, education level, and annual income [Wilk’s λ = 0.78, *F*(8,1596) = 26.36, *p* < 0.001, η^2^ = 0.22]. Follow-up univariate Analysis of Variance tests (ANOVAs) with Tukey’s Honest Significant Difference (HSD) *post hoc* tests are presented in Table 5. The *Selfless*, *Selfless Materialist*, and *Interdependent Insider* clusters have statistically equivalent below-average levels of depressive symptoms and neuroticism. The *Self-centered* and *Self-centered Non-materialist* clusters have statistically different above-average levels of depressive symptoms and neuroticism. The *Self-centered* cluster has the highest levels of depressive symptoms and neuroticism among all the clusters.

I conducted statistical tests to assess whether differences existed between clusters on demographic and control variables. Chi square yielded no statistically significant differences between clusters in gender or race, but differences in geographic location emerged (χ^2^ = 88.7, *p* < 0.001). For instance, participants who were born in Irvine, CA, United States were overrepresented in the *Selfless Materialist* cluster, whereas participants from Little Rock, AR, United States were underrepresented in the *Self-centered Non-materialist* cluster. One-way ANOVAs found no statistically significant differences in annual income between clusters, but differences did exist for educational level [*F*(4,805) = 2.65, *p* = 0.033, η^2^ = 0.01]. The *Self-centered Non-materialist* cluster had the highest average education level, whereas *Interdependent Insider* and *Self-Centered* clusters had the lowest education level.

### Discussion

The person-centered analyses identified multiple combinations of self-structures held by participants in a diverse sample. Average cluster-level differences in depressive symptoms and neuroticism validated the relationships established in the previous variable-centered analyses. *Selfless* and *Self-centered* clusters manifested as predicted by the current model of selflessness (Dambrun, 2017), but the majority of individuals held a configuration that included features of both selflessness and self-centeredness, providing evidence that a linear spectrum between selflessness and self-centeredness may not be the most meaningful conceptualization. This diverges from the usage of the terms in existing literature (e.g., Sonne and Gash, 2018).

Considering the combinations that did *not* emerge may be equally informative. The high degree of contrast in the levels of the three cluster indicators suggest that each of the dimensions may develop relatively independent from one another. One exception is the association between self-transcendence and perspective-taking; only one out of the five clusters (*Interdependent Insider*) exhibited discrepancies in the direction of self-transcendence and perspective-taking, suggesting that these factors tend to hang together, despite a low correlation (*r* = 0.019, *p* < 0.001). This is consistent with theoretical work that suggests perspective-taking is associated with the development of self-transcendence (Stewart and McHugh, 2013), as well as empirical studies that have found self-transcendent values to be compatible with perspective-taking (Silfver et al., 2008). More broadly, the diversity of cluster profiles suggests that there may be a variety of mechanisms which organize the structures of the self.

Consistent with previous variable-centered studies relating self-centeredness to well-being using the same theoretical approach (Dambrun et al., 2012; Dambrun, 2016, 2017; Hanley et al., 2017), the *Self-centered* cluster was found to have the highest levels of depressive symptoms and neuroticism. The *Selfless* cluster was expected to uniquely have the lowest measures of psychopathology, but this was not the case. Despite having lower values of perspective-taking and higher values of materialism than the *Selfless* cluster, the *Interdependent Insider* cluster has statistically equivalent levels (and higher absolute values) of depressive symptoms and neuroticism.

The associations between cluster membership and depressive symptoms and neuroticism appear to be driven by self-transcendence; all three clusters with above-average self-transcendence have the lowest values of depressive symptoms and neuroticism, whereas the two clusters with below-average self-transcendence have the highest values of depressive symptoms and neuroticism. This is consistent with the previous variable-centered analyses. The substantial negative correlation between self-transcendence and depressive symptoms (*r* = −0.47, *p* < 0.001) may overshadow the influence of perspective-taking and materialism (see Table 2). More broadly, the results suggest that some structures of the self (e.g., interdependence/independence, as measured by self-transcendence) may be more strongly related to psychopathological outcomes than other structures. Self-transcendence may be implicated in Dambrun and Ricard’s (2011) premise that the self-centeredness is primarily related to negative psychological well-being through cycles of approach and avoidance in response to pleasurable and painful stimuli (“hedonic principle”; also see Shiah, 2016).

## General Discussion

The multiple regression analyses found a significant interaction between perspective-taking and self-transcendence in relation to depressive symptoms. The cluster analysis identified five distinct profiles of self-structures in the sample that are differentially associated with depressive symptoms and neuroticism. The relationships between the clusters and depressive symptoms was driven by self-transcendence, further validating the findings of the regression analyses. Together, these two approaches demonstrate the usefulness of multidimensional perspectives in self psychology research.

The present work is aligned with dynamic-interactionist approaches toward studying the self, which emphasize the complex interdependence of the components of the self, interpersonal relationships, environmental features, and psychopathological outcomes (Zuroff, 1992; Zuroff et al., 2004; also see Kopala-Sibley and Zuroff, 2019 for a comparative description). Person-centered approaches provide a holistic account of multiple individual-level factors (Bergman et al., 2003).

The current study advances understanding of depression by framing psychopathology in relation to the structure of the self. Although this conceptualization has been previously suggested (Campbell et al., 2003; Stopa et al., 2010), the present research is the first to propose a link between the structure of the self and depression without invoking self-evaluation or information availability. The current study also provides a bridge between diverse literatures of selflessness, self-structure, and depression.

Beyond validating and complicating Dambrun and Ricard’s (2011) model, the results of the study contribute to a growing literature on selflessness and the relationships between selflessness and positive psychological outcomes (Haugan and Innstrand, 2012; Wayment et al., 2015; Hanley et al., 2017). Similar profiles of selflessness and self-centeredness may emerge in other multidimensional frameworks, especially those which rely on comparable measures (e.g., the Quiet Ego Scale; Wayment et al., 2015). The present study found that interdependence of the self (measured by self-transcendence) strongly predicted away from psychopathological outcomes; the relative dominance of this factor may also have implications in other frameworks. In light of the diverse profiles of self-structures that emerged from the cluster analyses, there is a need to examine the “hedonic principle” and explore other potential mechanisms that relate self-centeredness to negative psychological outcomes. The dataset utilized in the study is longitudinal, following participants from birth to age 26; future studies may use the data to investigate the antecedents of psychopathology and selflessness.

Measuring features of the self with self-report items is inherently fraught with epistemological challenges (Paulhus and Vazire, 2007; Grossman, 2008; Bergomi et al., 2013). A considerable weakness of the present paper is that the structures of the self are not directly measured, which constrains the types of conclusions that can be drawn. Although the inventories chosen to represent the dimensions of the self were consistent with previous research in the field (Dambrun et al., 2012; Dambrun, 2017; Hanley et al., 2017), the self-report data assesses self-knowledge and beliefs, which may not capture underlying self-structures. For instance, materialism may only partially capture the variance associated with underlying impermanence of the self. Further, self-report survey inventories can be problematic when assessing abstract self-constructs and may be susceptible to various forms of bias (e.g., response bias). Recently, measurements of selflessness have been developed utilizing experiential and implicit association methods (Hadash and Bernstein, 2019) and direct neurological assessment techniques (Farb et al., 2007; Dor-Ziderman et al., 2013). More broadly, future work on selflessness and depression would benefit from increased integration of methods and findings from neuroscience (Kopala-Sibley and Zuroff, 2019).

This paper is the latest in a line of inquiry that demonstrates the utility of integrating perspectives from Buddhism into psychological research (Olendzki, 2010; Segal and Teasdale, 2013; Shonin et al., 2014). The variable-centered analyses of the dimensions of the self yielded findings that contribute to separate literatures on self-transcendence and perspective-taking. Generally, the present study begins to clarify the relationship between the Buddhist notion of selflessness and positive psychological outcomes (Aronson, 2004; Hanley et al., 2017). Framing selflessness in terms of structure of the self frees the concept from Western connotations of altruism, self-negation, and withdrawal (Michalon, 2001; MacKenzie, 2016). Notwithstanding its limitations, the present study suggests that the relationship between selflessness and depression may be a fruitful direction for future research.

## Data Availability Statement

The datasets generated for this study are available on request to the corresponding author.

## Ethics Statement

The studies involving human participants were reviewed and approved by Institutional Review Board of the University of California, Irvine. The patients/participants provided their written informed consent to participate in this study.

## Author Contributions

The author confirms being the sole contributor of this work and has approved it for publication.

## Conflict of Interest

The author declares that the research was conducted in the absence of any commercial or financial relationships that could be construed as a potential conflict of interest.

## Appendix

### Depressive Symptoms (α = 0.94)

#### Instructions

Below is a list of the ways you might have felt or behaved. Please tell us how often you have felt this way during the past week.

(1) Rarely or none of the time (less than 1 day), (2) some or a little of the time (1–2 days), (3) occasionally or a moderate amount of time (3–4 days), (4) most or all of the time (5–7 days).

(1) I was bothered by things that usually don’t bother me.

(2) I did not feel like eating; my appetite was poor.

(3) I felt that I could not shake off the blues even with help from my family or friends.

(4) I felt I was just as good as other people.

(5) I had trouble keeping my mind on what I was doing.

(6) I felt depressed.

(7) I felt that everything I did was an effort.

(8) I felt hopeful about the future.

(9) I thought my life had been a failure.

(10) I felt fearful.

(11) My sleep was restless.

(12) I was happy.

(13) I talked less than usual.

(14) I felt lonely.

(15) People were unfriendly.

(16) I enjoyed life.

(17) I had crying spells.

(18) I felt sad.

(19) I felt that people dislike me.

(20) I could not get “going.”

Note: Four items were reverse coded: “(4) I felt I was just as good as other people,” “(8) I felt hopeful about the future,” “(12) I was happy,” and “(16) I enjoyed life.”

### Neuroticism (*r* = 0.64)

#### Instructions

Please tell us whether each of these describes you…

[(1) Not at all, (2) A little, (3) Some, (4) A lot]

(1) Worries a lot.

(2) Gets nervous easily.

### Self-Transcendence (α = 0.74)

#### Instructions

Please indicate how much you agree or disagree with the following statements.

[(1) Strongly disagree, (2) somewhat disagree, (3) neither agree nor disagree, (4) somewhat agree, (5) strongly agree]

(1) I often engage in quiet contemplation.

(2) I feel that my individual life is a part of a greater whole.

(3) My peace of mind is not easily upset.

(4) My happiness is not dependent on other people and things.

(5) I feel a sense of belonging with both earlier and future generations.

(6) I don’t worry about other people’s opinions of me.

Note: The first item, “I often engage in quiet contemplation,” exhibited low item-test correlation and was excluded from analyses.

### Perspective-Taking (α = 0.75)

#### Instructions

Please read the following statements then indicate how much you agree or disagree.

[(1) Strongly disagree, (2) disagree, (3) neither agree nor disagree, (4) agree, (5) strongly agree]

(1) Before criticizing somebody, I try to imagine how I would feel if I were in their place.

(2) When I’m upset at someone, I usually try to put myself in his or her shoes for a while.

(3) I try to look at everybody’s side of a disagreement before I make a decision.

(4) I sometimes find it difficult to see things from another person’s point of view.

(5) I believe that there are two sides to every question and try to look at them both.

Note: The fourth item, “I sometimes find it difficult to see things from another person’s point of view,” was reverse-coded.

### Materialism (α = 0.82)

#### Instructions

Please read the following statements then indicate how much you agree or disagree.

[(1) Strongly disagree, (2) disagree, (3) neutral, (4) agree, (5) strongly agree]

(1) I admire people who own expensive homes, cars, and clothes.

(2) The things I own say a lot about how well I’m doing in life.

(3) Buying things gives me a lot of pleasure.

(4) I like a lot of luxury in my life.

(5) My life would be better if I owned certain things I don’t have.

(6) I’d be happier if I could afford to buy more things.
