# Supplementary material for: Selflessness, Depression, and Neuroticism: An Interactionist Perspective on the Effects of Self-Transcendence, Perspective-Taking, and Materialism
Source: Front Psychol. 2020 Sep 23;11:523950. doi: 10.3389/fpsyg.2020.523950 (PMC7543651; doi:10.3389/fpsyg.2020.523950)
Supplement: Supplementary file 1 [file Table_1.DOCX]

********************************************************************************

********************************************************************************

** Supplementary materials for:

**

** Selflessness, Depression, and Neuroticism:

** An Interactionist Perspective on the Effects of Self-transcendence,

** Perspective-taking, and Materialism

********************************************************************************

********************************************************************************

********************************************************************************

// This document provides code that is intended to facilitate replication

// of the results of the study. The code was written in STATA for use with

// the NICHD SECCYD Age 26 dataset and NICHD SECCYD wave 1 dataset. Feel free

// to contact the author of the manuscript with questions.

****************************

** Process measures for analyses

****************************

// Collect variables needed from other datasets

///////////////////////////////////////////////

keep id Q51a Q51b Q51c Q51d Q51e Q51f /// ASTI

Q115a Q115b Q115c Q115d Q115e /// Perspective taking

Q116a Q116b Q116c Q116d Q116e Q116f /// Materialism

Q52a Q52b Q52c Q52d Q52e Q52f Q52g Q52h Q52i Q52j Q52k Q52l Q52m Q52n Q52o Q52p Q52q Q52r Q52s Q52t /// Depression

Q50e Q50j /// Neuroticism

cfemale cethnic site MEDUCM01 INCNTM01 /// Covariates from wave 1

salarytotal26 highdegree26 // Covariates from age 26

// Adult Self-Transcendence Inventory (ASTI): 51

///////////////////////////////////////////////

alpha Q51a Q51b Q51c Q51d Q51e Q51f, item

** Alpha is too low, the first item has to be dropped!

alpha Q51b Q51c Q51d Q51e Q51f, item

gen tempcount=0

gen tempsum=0

foreach i in b c d e f {

replace tempcount=tempcount+1 if Q51`i'!=.

replace tempsum=tempsum+Q51`i' if Q51`i'!=.

}

gen selftranscendence=.

replace selftranscendence=tempsum/tempcount if tempcount!=0

label variable selftranscendence "Self-transcendence - ASTI"

drop tempcount tempsum

// Davis Interpersonal Reactivity Index: 115

///////////////////////////////////////////////

gen tempRd=.

replace tempRd=6-Q115d if Q115d!=.

gen tempcount=0

gen tempsum=0

foreach i in a b c e {

replace tempcount=tempcount+1 if Q115`i'!=.

replace tempsum=tempsum+Q115`i' if Q115`i'!=.

}

replace tempcount=tempcount+1 if tempRd!=.

replace tempsum=tempsum+tempRd if tempRd!=.

gen perstaking=.

replace perstaking=tempsum/tempcount if tempcount!=0

label variable perstaking "Perspective-taking empathy - Davis IRI"

drop tempcount tempsum

alpha Q115a Q115b Q115c tempRd Q115e, item

drop tempRd

// Material Values Scale: 116

///////////////////////////////////////////////

gen tempcount=0

gen tempsum=0

foreach i in a b c d e f {

replace tempcount=tempcount+1 if Q116`i'!=.

replace tempsum=tempsum+Q116`i' if Q116`i'!=.

}

gen materialism=.

replace materialism=tempsum/tempcount if tempcount!=0

label variable materialism "Overall materialism - material values scale"

drop tempcount tempsum

alpha Q116a Q116b Q116c Q116d Q116e Q116f, item

// Center for Epidemiologic Studies Depression scale: 52

///////////////////////////////////////////////

gen tempcount=0

gen tempsum=0

replace Q52d=5-Q52d

replace Q52h=5-Q52h

replace Q52l=5-Q52l

replace Q52p=5-Q52p

foreach i in a b c d e f g h i j k l m n o p q r s t {

replace tempcount=tempcount+1 if Q52`i'!=.

replace tempsum=tempsum+Q52`i' if Q52`i'!=.

}

gen depression=.

replace depression=tempsum/tempcount if tempcount!=0

label variable depression "Depressive symptoms, CES-D"

replace Q52d=5-Q52d

replace Q52h=5-Q52h

replace Q52l=5-Q52l

replace Q52p=5-Q52p // Need to reset these to non-reverse coded

drop tempcount tempsum

alpha Q52a Q52b Q52c Q52d Q52e Q52f Q52g Q52h Q52i Q52j Q52k Q52l Q52m Q52n ///

Q52o Q52p Q52q Q52r Q52s Q52t, item

// Personality Assessment: 50

///////////////////////////////////////////////

gen neuroticism=.

replace neuroticism=(Q50e+Q50j)/2 if Q50e!=. & Q50j!=.

label variable neuroticism"Neuroticism personality domain"

alpha Q50e Q50j, item

pwcorr Q50e Q50j, sig

/// Summary

sum selftranscendence perstaking materialism depression neuroticism selftranscendence_all6

sum salarytotal26 highdegree26

tab cfemale

tab cethnic

sum MEDUCM01 INCNTM01

***************************

** Regressions and moderation tests

***************************

// Correlation tables

///////////////////////////////////////////////

** Use the actual dataset, NOT the imputed dataset!

// Generate dichotomous variables for the correlation table

gen white=.

replace white=0 if cethnic!=.

replace white=1 if cethnic==1

gen black=.

replace black=0 if cethnic!=.

replace black=1 if cethnic==2

gen hispanic=.

replace hispanic=0 if cethnic!=.

replace hispanic=1 if cethnic==3

gen other=.

replace other=0 if cethnic!=.

replace other=1 if cethnic==4

gen site0arkansas=.

replace site0arkansas=1 if site==0

replace site0arkansas=0 if site!=0

gen site1irvine=.

replace site1irvine=1 if site==1

replace site1irvine=0 if site!=1

gen site2kansas=.

replace site2kansas=1 if site==2

replace site2kansas=0 if site!=2

gen site3newhamp=.

replace site3newhamp=1 if site==3

replace site3newhamp=0 if site!=3

gen site4pitts=.

replace site4pitts=1 if site==4

replace site4pitts=0 if site!=4

gen site5philly=.

replace site5philly=1 if site==5

replace site5philly=0 if site!=5

gen site6virginia=.

replace site6virginia=1 if site==6

replace site6virginia=0 if site!=6

gen site7seattle=.

replace site7seattle=1 if site==7

replace site7seattle=0 if site!=7

gen site8northcaro=.

replace site8northcaro=1 if site==8

replace site8northcaro=0 if site!=8

gen site9wisconsin=.

replace site9wisconsin=1 if site==9

replace site9wisconsin=0 if site!=9

// Make correlation table

mkcorr selftranscendence perstaking materialism ///

depression neuroticism ///

cfemale white black hispanic other ///

highdegree26 salarytotal26 ///

site0arkansas site1irvine site2kansas site3newhamp site4pitts site5philly site6virginia site7seattle site8northcaro site9wisconsin, ///

log(correlation_table) replace sig means lab cdec(3) mdec(3)

// Regression models

///////////////////////////////////////////////

// Generate interaction variables

egen zselftranscendence=std(selftranscendence)

order zselftranscendence, after(selftranscendence)

egen zperstaking=std(perstaking)

order zperstaking, after(perstaking)

egen zmaterialism=std(materialism)

order zmaterialism, after(materialism)

gen selfXpers=selftranscendence*perstaking

gen selfXmat=selftranscendence*materialism

gen persXmat=perstaking*materialism

gen selfXpersXmat=selftranscendence*perstaking*materialism

gen zselfXpers=zselftranscendence*zperstaking

gen zselfXmat=zselftranscendence*zmaterialism

gen zpersXmat=zperstaking*zmaterialism

gen zselfXpersXmat=zselftranscendence*zperstaking*zmaterialism

// Depression outcome, standardized, short table

est clear

regress zdepression zselftranscendence zperstaking zmaterialism i.cfemale i.cethnic highdegree26 salarytotal26 i.site

est store m1

regress zdepression zselftranscendence zperstaking zmaterialism i.cfemale i.cethnic highdegree26 salarytotal26 i.site zselfXpers zselfXmat zpersXmat

est store m2

regress zdepression zselftranscendence zperstaking zmaterialism i.cfemale i.cethnic highdegree26 salarytotal26 i.site zselfXpers zselfXmat zpersXmat zselfXpersXmat

est store m3

esttab m* using depression_final_stand.csv, ///

cells(b(star fmt(3) label(Coef.)) se(par fmt(3) label(std.errors))) ///

title("Final_depression_regressions_stand") ///

replace label

// Neuroticism outcome, all variables standardized

est clear

regress zneuroticism zselftranscendence zperstaking zmaterialism i.cfemale i.cethnic highdegree26 salarytotal26 i.site

est store m1

regress zneuroticism zselftranscendence zperstaking zmaterialism i.cfemale i.cethnic highdegree26 salarytotal26 i.site zselfXpers zselfXmat zpersXmat

est store m2

regress zneuroticism zselftranscendence zperstaking zmaterialism i.cfemale i.cethnic highdegree26 salarytotal26 i.site zselfXpers zselfXmat zpersXmat zselfXpersXmat

est store m3

esttab m* using neuroticism_final_std.csv, ///

cells(b(star fmt(3) label(Coef.)) se(par fmt(3) label(std.errors))) ///

title("Final_neuroticism_regressions_standardized") ///

replace label

// Moderation graphs

///////////////////////////////////////////////

regress zdepression c.zselftranscendence##c.zperstaking zselftranscendence zperstaking zmaterialism zselfXmat zpersXmat zselfXpersXmat i.cfemale i.cethnic highdegree26 salarytotal26 i.site

margins, dydx(zselftranscendence) at(zperstaking=(-1(.1)1)) vsquish

margins, at(zselftranscendence=(-1 0 1) zperstaking=(-1(.1)1)) vsquish

marginsplot, x(zperstaking) recast(line) xlabel(-1(.1)1) level(`=round((normal(1)-normal(-1))*100,.01)') // Displays +/- 1 SE above and below

// 95% Confidence Interval: marginsplot, x(zperstaking) recast(line) xlabel(-1(.1)1)

// Putting noci takes away confidence intervals: marginsplot, noci x(zselftranscendence) recast(line) xlabel(-1(.1)1)

regress zdepression c.zperstaking##c.zselftranscendence zselftranscendence zperstaking zmaterialism zselfXmat zpersXmat zselfXpersXmat i.cfemale i.cethnic highdegree26 salarytotal26 i.site

margins, dydx(zperstaking) at(zselftranscendence=(-1(.1)1)) vsquish

margins, at(zperstaking=(-1 0 1) zselftranscendence=(-1(.1)1)) vsquish

marginsplot, x(zselftranscendence) recast(line) xlabel(-1(.1)1) level(`=round((normal(1)-normal(-1))*100,.01)') // Displays +/- 1 SE above and below

// 95% Confidence Interval: marginsplot, x(zselftranscendence) recast(line) xlabel(-1(.1)1)

// Putting noci takes away confidence intervals: marginsplot, noci x(zselftranscendence) recast(line) xlabel(-1(.1)1)

// Simple slopes

///////////////////////////////////////////////

regress depression c.selftranscendence##c.perstaking selftranscendence perstaking materialism selfXmat persXmat selfXpersXmat i.cfemale i.cethnic highdegree26 salarytotal26 i.site

estat vce

regress depression c.perstaking##c.selftranscendence selftranscendence perstaking materialism selfXmat persXmat selfXpersXmat i.cfemale i.cethnic highdegree26 salarytotal26 i.site

estat vce

regress zdepression c.zselftranscendence##c.zperstaking zselftranscendence zperstaking zmaterialism zselfXmat zpersXmat zselfXpersXmat i.cfemale i.cethnic highdegree26 salarytotal26 i.site

estat vce

regress zdepression c.zperstaking##c.zselftranscendence zselftranscendence zperstaking zmaterialism zselfXmat zpersXmat zselfXpersXmat i.cfemale i.cethnic highdegree26 salarytotal26 i.site

estat vce

gen selfXpers=selftranscendence*perstaking

gen selfXmat=selftranscendence*materialism

gen persXmat=perstaking*materialism

gen selfXpersXmat=selftranscendence*perstaking*materialism

gen zselfXpers=zselftranscendence*zperstaking

gen zselfXmat=zselftranscendence*zmaterialism

gen zpersXmat=zperstaking*zmaterialism

gen zselfXpersXmat=zselftranscendence*zperstaking*zmaterialism

regress depression c.selftranscendence##c.perstaking selftranscendence perstaking materialism selfXmat persXmat selfXpersXmat i.cfemale i.cethnic highdegree26 salarytotal26 i.site

margins, dydx(selftranscendence) at(perstaking=(-1(.1)1)) vsquish

margins, at(selftranscendence=(-1 0 1) perstaking=(-1(.1)1)) vsquish

marginsplot, noci x(perstaking) recast(line) xlabel(-1(.1)1)

regress depression c.perstaking##c.selftranscendence selftranscendence perstaking materialism selfXmat persXmat selfXpersXmat i.cfemale i.cethnic highdegree26 salarytotal26 i.site

margins, dydx(perstaking) at(selftranscendence=(-1(.1)1)) vsquish

margins, at(perstaking=(-1 0 1) selftranscendence=(-1(.1)1)) vsquish

marginsplot, noci x(selftranscendence) recast(line) xlabel(-1(.1)1)

***************************

** Cluster analyses

***************************

// Calculate mean-level differences

///////////////////////////////////////////////

** Cluster 1

estpost summarize depression neuroticism zdepression zneuroticism highdegree26 salarytotal26 MEDUCM01 INCNTM01 if five_cluster1==1

esttab using fivecluster_cluster1_cont.csv, cells("count mean sd min max") replace

putexcel set "fivecluster_cluster1_dich", modify

tab cfemale if cfemale==1 & five_cluster1==1, matcell(freq) matrow(names)

putexcel A2=("female") B2=matrix(freq)

tab cethnic if cethnic==1 & five_cluster1==1, matcell(freq) matrow(names)

putexcel A3=("white") B3=matrix(freq)

tab cethnic if cethnic==2 & five_cluster1==1, matcell(freq) matrow(names)

putexcel A4=("black") B4=matrix(freq)

tab cethnic if cethnic==3 & five_cluster1==1, matcell(freq) matrow(names)

putexcel A5=("hispanic") B5=matrix(freq)

tab site if site==0 & five_cluster1==1, matcell(freq) matrow(names)

putexcel A6=("site0arkansas") B6=matrix(freq)

tab site if site==1 & five_cluster1==1, matcell(freq) matrow(names)

putexcel A7=("site1irvine") B7=matrix(freq)

tab site if site==2 & five_cluster1==1, matcell(freq) matrow(names)

putexcel A8=("site2kansas") B8=matrix(freq)

tab site if site==3 & five_cluster1==1, matcell(freq) matrow(names)

putexcel A9=("site3newhamp") B9=matrix(freq)

tab site if site==4 & five_cluster1==1, matcell(freq) matrow(names)

putexcel A10=("site4pitts") B10=matrix(freq)

tab site if site==5 & five_cluster1==1, matcell(freq) matrow(names)

putexcel A11=("site5philly") B11=matrix(freq)

tab site if site==6 & five_cluster1==1, matcell(freq) matrow(names)

putexcel A12=("site6virginia") B12=matrix(freq)

tab site if site==7 & five_cluster1==1, matcell(freq) matrow(names)

putexcel A13=("site7seattle") B13=matrix(freq)

tab site if site==8 & five_cluster1==1, matcell(freq) matrow(names)

putexcel A14=("site8northcaro") B14=matrix(freq)

tab site if site==9 & five_cluster1==1, matcell(freq) matrow(names)

putexcel A15=("site9wisconsin") B15=matrix(freq)

** Cluster 2

estpost summarize depression neuroticism zdepression zneuroticism highdegree26 salarytotal26 MEDUCM01 INCNTM01 if five_cluster2==1

esttab using fivecluster_cluster2_cont.csv, cells("count mean sd min max") replace

putexcel set "fivecluster_cluster2_dich", modify

tab cfemale if cfemale==1 & five_cluster2==1, matcell(freq) matrow(names)

putexcel A2=("female") B2=matrix(freq)

tab cethnic if cethnic==1 & five_cluster2==1, matcell(freq) matrow(names)

putexcel A3=("white") B3=matrix(freq)

tab cethnic if cethnic==2 & five_cluster2==1, matcell(freq) matrow(names)

putexcel A4=("black") B4=matrix(freq)

tab cethnic if cethnic==3 & five_cluster2==1, matcell(freq) matrow(names)

putexcel A5=("hispanic") B5=matrix(freq)

tab site if site==0 & five_cluster2==1, matcell(freq) matrow(names)

putexcel A6=("site0arkansas") B6=matrix(freq)

tab site if site==1 & five_cluster2==1, matcell(freq) matrow(names)

putexcel A7=("site1irvine") B7=matrix(freq)

tab site if site==2 & five_cluster2==1, matcell(freq) matrow(names)

putexcel A8=("site2kansas") B8=matrix(freq)

tab site if site==3 & five_cluster2==1, matcell(freq) matrow(names)

putexcel A9=("site3newhamp") B9=matrix(freq)

tab site if site==4 & five_cluster2==1, matcell(freq) matrow(names)

putexcel A10=("site4pitts") B10=matrix(freq)

tab site if site==5 & five_cluster2==1, matcell(freq) matrow(names)

putexcel A11=("site5philly") B11=matrix(freq)

tab site if site==6 & five_cluster2==1, matcell(freq) matrow(names)

putexcel A12=("site6virginia") B12=matrix(freq)

tab site if site==7 & five_cluster2==1, matcell(freq) matrow(names)

putexcel A13=("site7seattle") B13=matrix(freq)

tab site if site==8 & five_cluster2==1, matcell(freq) matrow(names)

putexcel A14=("site8northcaro") B14=matrix(freq)

tab site if site==9 & five_cluster2==1, matcell(freq) matrow(names)

putexcel A15=("site9wisconsin") B15=matrix(freq)

** Cluster 3

estpost summarize depression neuroticism zdepression zneuroticism highdegree26 salarytotal26 MEDUCM01 INCNTM01 if five_cluster3==1

esttab using fivecluster_cluster3_cont.csv, cells("count mean sd min max") replace

putexcel set "fivecluster_cluster3_dich", modify

tab cfemale if cfemale==1 & five_cluster3==1, matcell(freq) matrow(names)

putexcel A2=("female") B2=matrix(freq)

tab cethnic if cethnic==1 & five_cluster3==1, matcell(freq) matrow(names)

putexcel A3=("white") B3=matrix(freq)

tab cethnic if cethnic==2 & five_cluster3==1, matcell(freq) matrow(names)

putexcel A4=("black") B4=matrix(freq)

tab cethnic if cethnic==3 & five_cluster3==1, matcell(freq) matrow(names)

putexcel A5=("hispanic") B5=matrix(freq)

tab site if site==0 & five_cluster3==1, matcell(freq) matrow(names)

putexcel A6=("site0arkansas") B6=matrix(freq)

tab site if site==1 & five_cluster3==1, matcell(freq) matrow(names)

putexcel A7=("site1irvine") B7=matrix(freq)

tab site if site==2 & five_cluster3==1, matcell(freq) matrow(names)

putexcel A8=("site2kansas") B8=matrix(freq)

tab site if site==3 & five_cluster3==1, matcell(freq) matrow(names)

putexcel A9=("site3newhamp") B9=matrix(freq)

tab site if site==4 & five_cluster3==1, matcell(freq) matrow(names)

putexcel A10=("site4pitts") B10=matrix(freq)

tab site if site==5 & five_cluster3==1, matcell(freq) matrow(names)

putexcel A11=("site5philly") B11=matrix(freq)

tab site if site==6 & five_cluster3==1, matcell(freq) matrow(names)

putexcel A12=("site6virginia") B12=matrix(freq)

tab site if site==7 & five_cluster3==1, matcell(freq) matrow(names)

putexcel A13=("site7seattle") B13=matrix(freq)

tab site if site==8 & five_cluster3==1, matcell(freq) matrow(names)

putexcel A14=("site8northcaro") B14=matrix(freq)

tab site if site==9 & five_cluster3==1, matcell(freq) matrow(names)

putexcel A15=("site9wisconsin") B15=matrix(freq)

** Cluster 4

estpost summarize depression neuroticism zdepression zneuroticism highdegree26 salarytotal26 MEDUCM01 INCNTM01 if five_cluster4==1

esttab using fivecluster_cluster4_cont.csv, cells("count mean sd min max") replace

putexcel set "fivecluster_cluster4_dich", modify

tab cfemale if cfemale==1 & five_cluster4==1, matcell(freq) matrow(names)

putexcel A2=("female") B2=matrix(freq)

tab cethnic if cethnic==1 & five_cluster4==1, matcell(freq) matrow(names)

putexcel A3=("white") B3=matrix(freq)

tab cethnic if cethnic==2 & five_cluster4==1, matcell(freq) matrow(names)

putexcel A4=("black") B4=matrix(freq)

tab cethnic if cethnic==3 & five_cluster4==1, matcell(freq) matrow(names)

putexcel A5=("hispanic") B5=matrix(freq)

tab site if site==0 & five_cluster4==1, matcell(freq) matrow(names)

putexcel A6=("site0arkansas") B6=matrix(freq)

tab site if site==1 & five_cluster4==1, matcell(freq) matrow(names)

putexcel A7=("site1irvine") B7=matrix(freq)

tab site if site==2 & five_cluster4==1, matcell(freq) matrow(names)

putexcel A8=("site2kansas") B8=matrix(freq)

tab site if site==3 & five_cluster4==1, matcell(freq) matrow(names)

putexcel A9=("site3newhamp") B9=matrix(freq)

tab site if site==4 & five_cluster4==1, matcell(freq) matrow(names)

putexcel A10=("site4pitts") B10=matrix(freq)

tab site if site==5 & five_cluster4==1, matcell(freq) matrow(names)

putexcel A11=("site5philly") B11=matrix(freq)

tab site if site==6 & five_cluster4==1, matcell(freq) matrow(names)

putexcel A12=("site6virginia") B12=matrix(freq)

tab site if site==7 & five_cluster4==1, matcell(freq) matrow(names)

putexcel A13=("site7seattle") B13=matrix(freq)

tab site if site==8 & five_cluster4==1, matcell(freq) matrow(names)

putexcel A14=("site8northcaro") B14=matrix(freq)

tab site if site==9 & five_cluster4==1, matcell(freq) matrow(names)

putexcel A15=("site9wisconsin") B15=matrix(freq)

** Cluster 5

estpost summarize depression neuroticism zdepression zneuroticism highdegree26 salarytotal26 MEDUCM01 INCNTM01 if five_cluster5==1

esttab using fivecluster_cluster5_cont.csv, cells("count mean sd min max") replace

putexcel set "fivecluster_cluster5_dich", modify

tab cfemale if cfemale==1 & five_cluster5==1, matcell(freq) matrow(names)

putexcel A2=("female") B2=matrix(freq)

tab cethnic if cethnic==1 & five_cluster5==1, matcell(freq) matrow(names)

putexcel A3=("white") B3=matrix(freq)

tab cethnic if cethnic==2 & five_cluster5==1, matcell(freq) matrow(names)

putexcel A4=("black") B4=matrix(freq)

tab cethnic if cethnic==3 & five_cluster5==1, matcell(freq) matrow(names)

putexcel A5=("hispanic") B5=matrix(freq)

tab site if site==0 & five_cluster5==1, matcell(freq) matrow(names)

putexcel A6=("site0arkansas") B6=matrix(freq)

tab site if site==1 & five_cluster5==1, matcell(freq) matrow(names)

putexcel A7=("site1irvine") B7=matrix(freq)

tab site if site==2 & five_cluster5==1, matcell(freq) matrow(names)

putexcel A8=("site2kansas") B8=matrix(freq)

tab site if site==3 & five_cluster5==1, matcell(freq) matrow(names)

putexcel A9=("site3newhamp") B9=matrix(freq)

tab site if site==4 & five_cluster5==1, matcell(freq) matrow(names)

putexcel A10=("site4pitts") B10=matrix(freq)

tab site if site==5 & five_cluster5==1, matcell(freq) matrow(names)

putexcel A11=("site5philly") B11=matrix(freq)

tab site if site==6 & five_cluster5==1, matcell(freq) matrow(names)

putexcel A12=("site6virginia") B12=matrix(freq)

tab site if site==7 & five_cluster5==1, matcell(freq) matrow(names)

putexcel A13=("site7seattle") B13=matrix(freq)

tab site if site==8 & five_cluster5==1, matcell(freq) matrow(names)

putexcel A14=("site8northcaro") B14=matrix(freq)

tab site if site==9 & five_cluster5==1, matcell(freq) matrow(names)

putexcel A15=("site9wisconsin") B15=matrix(freq)

// MANOVA/ANOVA tests

///////////////////////////////////////////////

**** Exploring MANOVA/MANCOVA

manova zdepression = fivecluster_unstd

manova zdepression zneuroticism = fivecluster_unstd

// MANCOVA

** The first is a test of equality of group effects adjusted for the covariates

manova zdepression zneuroticism = fivecluster_unstd cfemale cethnic c.highdegree26 c.salarytotal26

** Second, test that the coefficients for the covariates are jointly equal to zero

estat esize

manovatest cfemale cethnic c.highdegree26 c.salarytotal26

** Third, to test that the coefficients for the covariates are equal across groups, we perform a MANCOVA that includes our covariates (x1 and x2) interacted with group

manova zdepression zneuroticism = fivecluster_unstd cfemale cethnic c.highdegree26 c.salarytotal26 fivecluster_unstd#cfemale fivecluster_unstd#cethnic fivecluster_unstd#c.highdegree26 fivecluster_unstd#c.salarytotal26

manovatest fivecluster_unstd#cfemale fivecluster_unstd#cethnic fivecluster_unstd#c.highdegree26

mvreg

// Only for depression...

manova zdepression = fivecluster_unstd cfemale cethnic c.highdegree26 c.salarytotal26

mvreg

manovatest cfemale cethnic c.highdegree26 c.salarytotal26

// Only for neuroticism...

manova zneuroticism = fivecluster_unstd cfemale cethnic c.highdegree26 c.salarytotal26

mvreg

manovatest cfemale cethnic c.highdegree26 c.salarytotal26

**** Means across each cluster

sum zselftranscendence zperstaking zmaterialism if fivecluster_unstd==1

sum zdepression zneuroticism if fivecluster_unstd==1

sum highdegree26 salarytotal26 if fivecluster_unstd==1

tab cfemale if fivecluster_unstd==1

tab cethnic if fivecluster_unstd==1

tab site if fivecluster_unstd==1

sum zselftranscendence zperstaking zmaterialism if fivecluster_unstd==2

sum zdepression zneuroticism if fivecluster_unstd==2

sum highdegree26 salarytotal26 if fivecluster_unstd==2

tab cfemale if fivecluster_unstd==2

tab cethnic if fivecluster_unstd==2

tab site if fivecluster_unstd==2

sum zselftranscendence zperstaking zmaterialism if fivecluster_unstd==3

sum zdepression zneuroticism if fivecluster_unstd==3

sum highdegree26 salarytotal26 if fivecluster_unstd==3

tab cfemale if fivecluster_unstd==3

tab cethnic if fivecluster_unstd==3

tab site if fivecluster_unstd==3

sum zselftranscendence zperstaking zmaterialism if fivecluster_unstd==4

sum zdepression zneuroticism if fivecluster_unstd==4

sum highdegree26 salarytotal26 if fivecluster_unstd==4

tab cfemale if fivecluster_unstd==4

tab cethnic if fivecluster_unstd==4

tab site if fivecluster_unstd==4

sum zselftranscendence zperstaking zmaterialism if fivecluster_unstd==5

sum zdepression zneuroticism if fivecluster_unstd==5

sum highdegree26 salarytotal26 if fivecluster_unstd==5

tab cfemale if fivecluster_unstd==5

tab cethnic if fivecluster_unstd==5

tab site if fivecluster_unstd==5

**** Univariate ANOVAs and Tukey HSD tests

anova zselftranscendence fivecluster_unstd

estat esize

// You need to run the ANOVA/MANOVA in Stata before you can carry out post hoc tests

pwmean zselftranscendence, over(fivecluster_unstd) mcompare(tukey) effects

** pwmean DependentVariable, over[IndependentVariable] mcompare(tukey) effects

anova zperstaking fivecluster_unstd

estat esize

pwmean zperstaking, over(fivecluster_unstd) mcompare(tukey) effects

anova zmaterialism fivecluster_unstd

estat esize

pwmean zmaterialism, over(fivecluster_unstd) mcompare(tukey) effects

anova zdepression fivecluster_unstd

estat esize

pwmean zdepression, over(fivecluster_unstd) mcompare(tukey) effects

anova zneuroticism fivecluster_unstd

estat esize

pwmean zneuroticism, over(fivecluster_unstd) mcompare(tukey) effects

anova cfemale fivecluster_unstd

estat esize

pwmean cfemale, over(fivecluster_unstd) mcompare(tukey) effects

anova white fivecluster_unstd

estat esize

pwmean white, over(fivecluster_unstd) mcompare(tukey) effects

anova black fivecluster_unstd

estat esize

pwmean black, over(fivecluster_unstd) mcompare(tukey) effects

anova hispanic fivecluster_unstd

estat esize

pwmean hispanic, over(fivecluster_unstd) mcompare(tukey) effects

anova other fivecluster_unstd

estat esize

pwmean other, over(fivecluster_unstd) mcompare(tukey) effects

anova highdegree26 fivecluster_unstd

estat esize

pwmean highdegree26, over(fivecluster_unstd) mcompare(tukey) effects

anova salarytotal26 fivecluster_unstd

estat esize

pwmean salarytotal26, over(fivecluster_unstd) mcompare(tukey) effects

anova site0arkansas fivecluster_unstd

estat esize

pwmean site0arkansas, over(fivecluster_unstd) mcompare(tukey) effects

anova site1irvine fivecluster_unstd

estat esize

pwmean site1irvine, over(fivecluster_unstd) mcompare(tukey) effects

anova site2kansas fivecluster_unstd

estat esize

pwmean site2kansas, over(fivecluster_unstd) mcompare(tukey) effects

anova site3newhamp fivecluster_unstd

estat esize

pwmean site3newhamp, over(fivecluster_unstd) mcompare(tukey) effects

anova site4pitts fivecluster_unstd

estat esize

pwmean site4pitts, over(fivecluster_unstd) mcompare(tukey) effects

anova site5philly fivecluster_unstd

estat esize

pwmean site5philly, over(fivecluster_unstd) mcompare(tukey) effects

anova site6virginia fivecluster_unstd

estat esize

pwmean site6virginia, over(fivecluster_unstd) mcompare(tukey) effects

anova site7seattle fivecluster_unstd

estat esize

pwmean site7seattle, over(fivecluster_unstd) mcompare(tukey) effects

anova site8northcaro fivecluster_unstd

estat esize

pwmean site8northcaro, over(fivecluster_unstd) mcompare(tukey) effects

anova site9wisconsin fivecluster_unstd

estat esize

pwmean site9wisconsin, over(fivecluster_unstd) mcompare(tukey) effects

// I could have used ANOVA for all dichotomous variables, but this isn't

// easily interpretable and the standard approach is to use chi-square

// with categorical variables.

tabulate cfemale fivecluster_unstd, chi2

tabulate white fivecluster_unstd, chi2

tabulate black fivecluster_unstd, chi2

tabulate hispanic fivecluster_unstd, chi2

tabulate other fivecluster_unstd, chi2

tabulate site0arkansas fivecluster_unstd, chi2

tabulate site1irvine fivecluster_unstd, chi2

tabulate site2kansas fivecluster_unstd, chi2

tabulate site3newhamp fivecluster_unstd, chi2

tabulate site4pitts fivecluster_unstd, chi2

tabulate site5philly fivecluster_unstd, chi2

tabulate site6virginia fivecluster_unstd, chi2

tabulate site7seattle fivecluster_unstd, chi2

tabulate site8northcaro fivecluster_unstd, chi2

tabulate site9wisconsin fivecluster_unstd, chi2
